# Exosome-Based Theranostics for Liver Diseases

**DOI:** 10.1155/2022/7888906

**Published:** 2022-11-02

**Authors:** Nianan Luo, Jiangbin Li, Rui Dong, Jianguo Lu

**Affiliations:** ^1^Tangdu Hospital, Fourth Military Medical University, Xi'an 710038, China; ^2^943 Hospital of PLA, Wuwei 733000, China

## Abstract

Exosomes are small extracellular vesicles that can be secreted by any type of cell, released into almost all biological fluids, and extracted from anybody fluid such as blood, urine, saliva, and amniotic fluid. The theranostic role of exosome in liver diseases has been widely studied in recent years. In this review, we briefly introduce the biological characteristics of exosomes and then focus on the theranostics of exosomes in liver diseases, specifically gene delivery associated with liver diseases.

## 1. Introduction

Liver disease is the total of all diseases that happen in liver, including infectious disease, oncologic disease, vascular disease, metabolic disease, toxic disease, autoimmune disease, hereditary disease, and stone disease of bile duct inside liver [1–3]. Liver disease can lead to abnormal liver function and even liver failure [4–7].

Exosomes were first discovered by Johnstone [8]. In 1987, they defined these functional vesicles as exosomes [9]. In recent years, exosomes have attracted more and more attention from the scientific community, and they have gradually become a hot spot in the field of medical research [10]. Exosomes can carry a series of complex bioactive substances, which are important mediators of intercellular signal transduction, and exert their biological effects through complex mechanisms [11, 12]. Because of the specificity of exosomes' source and composition, they may indirectly reflect the underlying pathological changes in patients, and therefore may represent a new biomarker for the diagnosis and evaluation of clinically relevant diseases [13, 14]. In addition, much effort has been devoted to exosomes as drug delivery systems for the treatment of diseases [15]. The discovery of different types of exosomes, and the transport of these exosomes to tissues and organs in the body, completing cell-to-cell communication, has prompted researchers to focus on exosomes' therapeutic potential, particularly as drug carriers [16]. To date, cell-derived exosomes have shown multiple advantages over other existing or potential drug delivery vectors, including natural composition, small size (nanoscale), and immune invisibility [17–19]. The roles of exosomes in cardiovascular, cancer, diabetes, and other diseases has been well-demonstrated [13, 20, 21], and their biological characteristics may bring new ideas for treating liver diseases.

In this review, we focus on the roles of exosomes in liver diseases, including diagnostic biomarkers and therapeutic delivery vectors for liver diseases.

## 2. Characteristics of Exosomes

### 2.1. Composition of Exosomes

Exosomes are cell-derived vesicles, approximately 50-150 nm in diameter, with endosomal origin and inheriting the phospholipid membrane of the parental cell [22]. Notably, their biochemical components include not only lipids and proteins but also nucleic acids such as miRNAs and mRNA, and the presence of genomic and mitochondrial DNA has even been reported [23]. These bioactive substances are important mediators of intercellular signal transduction. Exosomes regulate recipient cells through information exchange between cells and are widely involved in the physiological and pathological processes of human body, such as immune regulation, cell proliferation and differentiation, and tumor invasion and metastasis [24, 25]. CD63 and Tsg101 proteins are the most specific proteins in exosomes, CD9 and CD81 proteins are the most common proteins in exosomes, and exosomes are also rich in CD8 and Alix proteins. These proteins provide a basis for the identification and engineering of exosomes [26].

### 2.2. Exosomes and Gene Delivery

Exosomes, as natural gene delivery carriers, have the advantages of stability, safety and targeting [27, 28]. Their nonviral characteristics give them high biocompatibility, low clearance rate, and suitability for targeted cell delivery. By fusing with viral vectors, exosomes significantly reduce the immunogenicity and virulence of viral vectors and reduce the rapid clearance and long-term humoral response of viral vectors. In addition, exosomes can mediate the delivery of macromolecular mRNA, carrying functional mRNA, and delivering them to target cells or tissues [29]. Engineered exosomes can carry large DNA molecules, such as CRISPR/Cas9 expression plasmids, for targeted delivery to specific cells or tissues [30]. Exosomes can be engineered by fusing surface proteins CD63, CD9 and lysosomal associated membrane proteins 2b (Lamp2b) with target cell peptides to increase the loading efficiency and capacity of exosomes and enable selective targeting of exosomes to avoid unnecessary accumulation in other organs, thereby reducing systemic toxicity [31].

## 3. Biological Functions of Exosomes in Liver Diseases

### 3.1. Exosome-Based Diagnosis

Exosomes are mainly composed of proteins and lipids; however, their main functions are likely to be achieved by small ncRNA, mRNA, DNA, and proteins that carry molecular information between cells. Recently, Zomer et al. found that purified exosomes contain functional miRNAs and small ncRNA, but mRNA is rarely detected [32]. The cell type or source of exosomes determines their composition, function, and molecular information [26], and they can carry different information to different cells due to the composition of exosomes [33]. Clinically, liver biopsy is the gold standard for the diagnosis of benign and malignant liver tumors, but biopsy is invasive, expensive, and has certain risks for patients.

**Figure 1:**
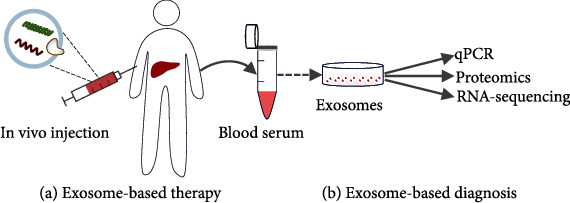
Exosome-based theranostics for liver diseases. (a) The native or bioengineered exosomes is injected for liver disease therapy in vivo. (b) Exosomes from different liver cell types are isolated for liver diseases diagnosis.

Exosomes can be used in liquid biopsy for the diagnosis of benign and malignant liver tumors. Wang et al. reported that exosomes play a key role in the diagnosis of hepatocellular carcinoma (HCC) [34]. Zhang et al. found that exosome-delivered epidermal growth factor receptor (EGFR) regulates liver microenvironment to promote gastric cancer liver metastasis [35]. Zhao et al. discovered that serum-derived exosomal proteins as potential candidate biomarkers for HCC [36]. Exosomes can indirectly reflect the pathological state of the liver. Therefore, they can be used in the diagnosis and evaluation of liver disease (Figure 1).

### 3.2. Exosome-Based Therapy

The bioactivity of exosomes holds great promise for the treatment of liver diseases [34, 37, 38]. It has been demonstrated that some bioactive substances can interact with each other through exosomes. For example, exosomes derived from human amniotic epithelial cells significantly reduce the number and infiltration of macrophages. In recent years, more and more studies have been conducted on exosomes and liver diseases. It has been found that exosomes in healthy subjects contain a variety of molecules that interact with and alter extracellular matrix (ECM) components, including matrix metalloproteinase-1 (MMP-1), insulin-degrading enzyme, heparinase, and integrin [39]. These enzymes can potentially locate on the surface of exosomes through contact with molecules in ECM, resulting in the cleavage of substrates such as collagen. Fibronectin-rich exosomes interact with integrins to promote cell adhesion [40]. Therefore, exosomes are involved in the regulation of liver inflammation and ECM remodeling.

It has been reported that quiescent hepatic stellate cells- (HSCs-) exosomes can inhibit the activation of HSCs and block the pathway of liver fibrosis formation, allowing HSCs to return to a more quiescent phenotype [41]. Hepatocyte-derived exosomes have also been shown to reverse the expression of fibrosis related genes and ethanol-induced hepatocyte injury [42, 43]. Therefore, exosomes produced by quiescent HSCs or normal hepatocytes may play an important role in reducing the progression of fibrosis and have great potential for antifibrotic treatment. Exosome as a carrier for the treatment of liver fibrosis is rarely reported, and its role in fibrogenesis and as a treatment for reversing fibrosis deserves further investigation.

## 4. Conclusions and Prospective

Cell-derived exosomes carry a series of complex bioactive substances, which are important mediators of intercellular signal transduction and are widely involved in regulating the physiological and pathological processes such as immune response, cell function, and tumor invasion and metastasis [44]. Exosomes possess stability, safety, and targeting properties that nonviral vectors lack [45]. Surface engineering of exosomes can increase the concentration of local exosomes, thereby reducing the toxicity and side effects and maximizing therapeutic effects [46]. Exosomes have been used for drug delivery and transport of various substances. Current methods of drug loading by exosomes include electroporation, incubation, or transfection of source cells [47]. Catalase has also been reported to be loaded into exosomes by ultrasound and extrusion or osmosis with saponin, which is more efficient than simple incubation [48]. Most studies have shown that exosomes could load and exchange small nucleic acids (miRNAs and siRNA) to target cells and organs and exhibit therapeutic effects. Other substances such as proteins and small molecules have also been reported. However, due to the small volume of exosomes, their loading of macromolecules is worthy of further study.

Although exosomes are natural vectors, they can be easily modified by surface modification, which aims to give them cell targeting specificity. The modification strategies mainly include genetic engineering and chemical modification [46]. Exosome genetic engineering is a convenient way to give exosomes new properties by fusing the gene sequence of a guide protein or peptide with the gene sequence of a selected exosome membrane protein [47]. In the genetic engineering approach, the targeted ligand or peptide is fused with exosome surface transmembrane proteins, and then donor cells encoding the fusion protein granules are transfected to secrete engineered exosomes carrying the targeted ligand on the surface [49]. Exosome gene engineering represents the strategy of exosome targeted modification, but it requires plasmid construction and protein overexpression in donor cells [45]. Lipid or biological coupling reactions can also anchor targeted substances to exosomal membranes. Chemical methods rely on biological coupling of targeted ligands to surface proteins, but inactivation of surface proteins or accumulation of exosomes may occur during chemical operations. Although these methods have certain limitations, they have been successfully implemented [30, 31, 50]. Exosomes as natural gene delivery vectors have been well studied and their versatility, reengineering, and ability to cross biological barriers have been proven [51]. However, poor targeting remains a major obstacle to their potential application. The biological characteristics of the exosome make it a diagnostic biomarker and a safe and efficient gene delivery vector, which may bring new ideas for the diagnosis and treatment of liver diseases.s

## Data Availability

All data generated or analyzed during this study are included in the current article.
